# Cochlear Implantation Outcomes in Patients with Auditory Neuropathy Spectrum Disorder of Genetic and Non-Genetic Etiologies: A Multicenter Study

**DOI:** 10.3390/biomedicines10071523

**Published:** 2022-06-28

**Authors:** Pei-Hsuan Lin, Hung-Pin Wu, Che-Ming Wu, Yu-Ting Chiang, Jacob Shujui Hsu, Cheng-Yu Tsai, Han Wang, Li-Hui Tseng, Pey-Yu Chen, Ting-Hua Yang, Chuan-Jen Hsu, Pei-Lung Chen, Chen-Chi Wu, Tien-Chen Liu

**Affiliations:** 1 Graduate Institute of Clinical Medicine, College of Medicine, National Taiwan University, Taipei 10002, Taiwan; peihsuanlin@ntu.edu.tw (P.-H.L.); paylong@ntu.edu.tw (P.-L.C.); 2 Department of Otolaryngology, National Taiwan University Hospital, Taipei 10002, Taiwan; d10455001@ntu.edu.tw (Y.-T.C.); d06455008@ntu.edu.tw (C.-Y.T.); 117887@ntuh.gov.tw (H.W.); 101975@ntuh.gov.tw (L.-H.T.); thyang37@ntu.edu.tw (T.-H.Y.); cjhsu@ntu.edu.tw (C.-J.H.); 3 Department of Otolaryngology, National Taiwan University Hospital Yunlin Branch, Yunlin 64041, Taiwan; 4 Department of Otolaryngology, Taichung Tzu Chi Hospital, Buddhist Tzu Chi Medical Foundation, Taichung 42743, Taiwan; hungpin_wu@tzuchi.com.tw; 5 Department of Otolaryngology & Head and Neck Surgery, New Taipei Municipal TuCheng Hospital (Built and Operated by Chang Gung Medical Foundation), New Taipei City 23652, Taiwan; bobwv@cgmh.org.tw; 6 Department of Otolaryngology & Head and Neck Surgery, Chang Gung Memorial Hospital, Chang Gung University, Linkou, Taoyuan 33305, Taiwan; 7 Graduate Institute of Medical Genomics and Proteomics, College of Medicine, National Taiwan University, Taipei 10051, Taiwan; jacobhsu@ntu.edu.tw; 8 Department of Otolaryngology, MacKay Memorial Hospital, Taipei 10449, Taiwan; pyc.4611@mmh.org.tw; 9 Department of Audiology and Speech-Language Pathology, Mackay Medical College, New Taipei City 25245, Taiwan; 10 Department of Medical Genetics, National Taiwan University Hospital, Taipei 10002, Taiwan; 11 Department of Medical Research, National Taiwan University Hospital Hsin-Chu Branch, Hsin-Chu 30261, Taiwan; 12 Department of Otolaryngology, National Taiwan University Hospital Hsin-Chu Branch, Hsin-Chu 30261, Taiwan; 13 Hearing and Speech Center, National Taiwan University Hospital, Taipei 10002, Taiwan

**Keywords:** auditory neuropathy spectrum disorder, *OTOF*, *WFS1*, *OPA1*, cochlear nerve deficiency, cochlear implant, outcome

## Abstract

With diverse etiologies and clinical features, the management of pediatric auditory neuropathy spectrum disorder (ANSD) is often challenging, and the outcomes of cochlear implants (CIs) are variable. This study aimed to investigate CI outcomes in pediatric patients with ANSD of different etiologies. Thirty-six children with ANSD who underwent cochlear implantation between 2001 and 2021 were included. Comprehensive etiological analyses were conducted, including a history review, next-generation sequencing-based genetic examinations, and imaging studies using high-resolution computed tomography and magnetic resonance imaging. Serial behavioral and speech audiometry were performed before and after surgery, and the outcomes with CI were evaluated using the Categories of Auditory Performance (CAP) and Speech Intelligibility Rating (SIR) scores. By etiology, 18, 1, 1, and 10 patients had *OTOF*-related, *WFS1*-related, *OPA1*-related, and cochlear nerve deficiency (CND)-related ANSD, respectively. Six patients had no definite etiology. The average CI-aided behavioral threshold was 28.3 ± 7.8 dBHL, and those with CND-related ANSD were significantly worse than *OTOF*-related ANSD. The patients’ median CAP and SIR scores were 6 and 4, respectively. Favorable CI outcomes were observed in patients with certain etiologies of ANSD, particularly those with *OTOF* (CAP/SIR scores 5–7/2–5), *WFS1* (CAP/SIR score 6/5), and *OPA1* variants (CAP/SIR score 7/5). Patients with CND had suboptimal CI outcomes (CAP/SIR scores 2–6/1–3). Identifying the etiologies in ANSD patients is crucial before surgery and can aid in predicting prognoses.

## 1. Introduction

Auditory neuropathy spectrum disorder (ANSD) is an important entity in pediatric sensorineural hearing loss (SNHL), accounting for approximately 10% of cases [1,2]. The etiologies of ANSD are heterogeneous; both genetic causes (e.g., *OTOF* and *PJVK* variants) and acquired risk factors (e.g., prematurity, kernicterus, perinatal hypoxia) can contribute to ANSD [3,4]. ANSD is characterized by impaired or absent auditory brainstem responses (ABRs) with preserved otoacoustic emissions (OAEs) and/or cochlear microphonics (CMs) [4,5]. The pathogenesis of ANSD encompasses a wide range of disease mechanisms, and ANSD pathologies can be localized to multiple sites, from the inner hair cells to the central auditory cortex [3].

The audiologic results often change during different audiometric examinations with relatively poor correlations [6]. The clinical presentations of ANSD patients are diverse, ranging from mild to profound SNHL [7], and the thresholds may fluctuate between different examinations [4]. ANSD patients generally present disproportionately poor speech recognition and language development [2,4], although approximately 5% of ANSD patients reportedly develop normal speech and language performance without intervention [8]. Speech performance is highly variable even with the use of hearing aids or cochlear implants (CIs) [9,10,11]. Therefore, the management of ANSD is often challenging in clinical practice [7].

Accumulating evidence reveals that CI outcomes correlate closely with the etiologies of pediatric SNHL [12,13,14,15]. We recently demonstrated that comprehensive history-taking, genetic examinations, and imaging studies were useful in addressing the etiological heterogeneity of pediatric ANSD and could help obtain information regarding the causes of ANSD in 75% of the patients [6]. In this study, we applied this integrative approach to analyze the etiologies of CI in recipients with pediatric ANSD and investigated the CI outcomes across various etiologies.

## 2. Materials and Methods

ANSD patients who had undergone cochlear implantation at three referral CI centers from 2001 to 2021 were enrolled. All patients were diagnosed with ANSD based on audiometric presentations: bilateral SNHL with absent ABRs and preserved OAEs and/or CMs. Comprehensive etiological analyses were performed for all patients, including history ascertainment, genetic examinations, and imaging studies. There was no patient with unilateral SNHL or cognitive comorbidities. All patients received complete etiological analyses, and one patient with follow-up less than 6 months after cochlear implantation was excluded.

### 2.1. Genetic Examinations

All patients underwent next-generation sequencing (NGS)-based genetic examination targeting 220 deafness genes. Genomic DNA was extracted from the peripheral blood of the patients to generate DNA libraries. Sample preparation, DNA sequencing, and NGS-based genetic testing data analyses were performed as previously described [16,17]. Paired-end reads were aligned, sorted, and converted using Picard version 1.134 (Broad Institute, Cambridge, MA, USA) and BWA-MEM version 0.7.12. Variants, including single nucleotide substitutions and small deletions/insertions, were called GATK HaplotypeCaller version 3.6 (Broad Institute, Cambridge, MA, USA). The pathogenicity of the variants was determined according to American College of Medical Genetics and Genomics guidelines. Variants that met the criteria of pathogenic/likely pathogenic were reported as disease-causing, and those with conclusive genetic diagnosis were confirmed to have genetic causes.

### 2.2. Imaging Studies

Both temporal bone high-resolution computed tomography (HRCT) and non-contrast brain magnetic resonance imaging (MRI) were performed preoperatively in all patients to evaluate the inner ear structures, cochlear nerve, and central auditory pathway [6]. Abnormalities on HRCT or MRI images were interpreted according to the published criteria [18,19]. Cochlear nerve deficiency (CND) was defined if the cochlear nerve was hypoplastic (the diameter of the cochlear nerve being smaller than the facial nerve on oblique sagittal MRI in the internal auditory canal) or aplastic (absent cochlear nerve on MRI) [18].

### 2.3. CI Outcome Evaluation

We performed serial behavioral and speech audiometry before and after cochlear implantation according to the patients’ age and speech development. Audiologic examinations were performed at 1, 3, 6, 9, and 12 months in the first year postoperatively. From the second year, examinations were performed every 6 months or whenever indicated. Hearing thresholds were calculated as the average of the four frequencies (0.5, 1, 2, and 4 kHz). The Categories of Auditory Performance (CAP) [20] and Speech Intelligibility Rating (SIR) [21] scores were evaluated whenever suitable for the patients. CI outcomes were determined using behavioral audiometry, speech audiometry, and CAP and SIR scores. According to our in-house data on Taiwanese patients, favorable CI outcomes were determined in those with CAP scores ≥ 5 or SIR scores ≥ 3, whereas unfavorable CI outcomes were determined in those with CAP scores < 5 and SIR scores < 3 [13].

### 2.4. Statistical Analysis

Categorical data were analyzed using Fisher’s exact test, and both ordinal and continuous data were analyzed using the Mann–Whitney U test or Kruskal–Wallis test. Post hoc tests were conducted for multiple comparisons with Bonferroni correction. Statistical significance was set at *p* values < 0.05 (two-sided). All statistical analyses were performed using SPSS version 26 software (SPSS Inc., Chicago, IL, USA).

## 3. Results

A total of 36 patients (24 males and 12 females) were included in this study. Fourteen (38.9%) patients received bilateral CI implantation. By etiology, 20 patients were confirmed to have a conclusive genetic diagnosis (Table 1). Of these, 18 patients (50%) were diagnosed with biallelic *OTOF* variants (i.e., the “*OTOF* variant” group). Pathogenic variants in two rare dominant genes were identified in two (5.6%) patients, including one with the *WFS1* c.2051C>T (p.A684V) variant and one with the *OPA1* c.1414T>C (p.C472R) variant, respectively (i.e., the “rare gene variants” group). Ten (27.8%) patients were diagnosed as having CND by MRI study (i.e., the “CND” group). Definite etiologies could not be identified in the remaining six patients based on the history review, genetic examinations, and imaging studies (i.e., the “indefinite” group). No anomalies in the inner ear other than the CND and no lesions in the brain were identified.

The average age at implantation of the first ear was 3.9 ± 4.9 (1–27.7) years (Table 2), probably reflecting the heterogenous etiologies and audiological features in our cohort. The preoperative hearing thresholds were significantly better in patients with rare gene variants (Kruskal–Wallis test, *p* = 0.022), probably because the retrocochlear nature of *OPA1* and *WFS1* variants led to disproportionately poor speech recognition, necessitating an earlier cochlear implantation at a better hearing level. Conversely, patients with CND showed the worst preoperative hearing thresholds (Bonferroni-adjusted *p* = 0.03). There was no significant difference in the average implantation age of the first ear, number of patients receiving bilateral implantation, and follow-up period among the four groups (*p* > 0.05).

After cochlear implantation, the average CI-aided hearing threshold in all 36 patients was 28.3 ± 7.8 (18.8–55) dBHL, and the median CAP and SIR scores were 6 and 4, respectively (Table 3). The average CI-aided hearing threshold in patients with CND was significantly higher (Kruskal–Wallis test, *p* < 0.001) and was especially higher than that in patients with *OTOF* variants and rare gene variants (Bonferroni-adjusted *p* = 0.003 and 0.001, respectively). Twenty-seven patients (75%) showed favorable CI outcomes with CAP ≥ 5 or SIR ≥ 3. Both CAP and SIR scores were significantly worse in the CND group than in the other groups (Kruskal–Wallis test, *p* = 0.021 and 0.006, respectively). Specifically, the CAP and SIR scores were significantly lower in the CND group than in the *OTOF* variant group (Bonferroni-adjusted *p* = 0.021 for CAP and *p* = 0.001 for SIR). Overall, the CI outcomes were good in most ANSD patients except in those with CND.

### 3.1. CI Outcomes in ANSD Patients with Biallelic OTOF Variants

Eighteen patients were confirmed to have biallelic *OTOF* variants, including seven with homozygous c.5098G>C variants and eleven with compound heterozygous variants. Nine (50%) patients underwent bilateral implantation, one of whom received bilateral simultaneous implantation. The preoperative hearing threshold was 88.2 ± 14.5 (57.5–106.3) dBHL, and the CI-aided hearing threshold was 25.6 ± 4.1 (20–35) dBHL. The median CAP and SIR scores at the latest follow-up were 7 (range, 5–7) and 5 (range, 2–5), respectively, indicating that these patients showed excellent outcomes.

### 3.2. CI Outcomes in ANSD Patients with Rare Gene Variants

**Figure 1:**
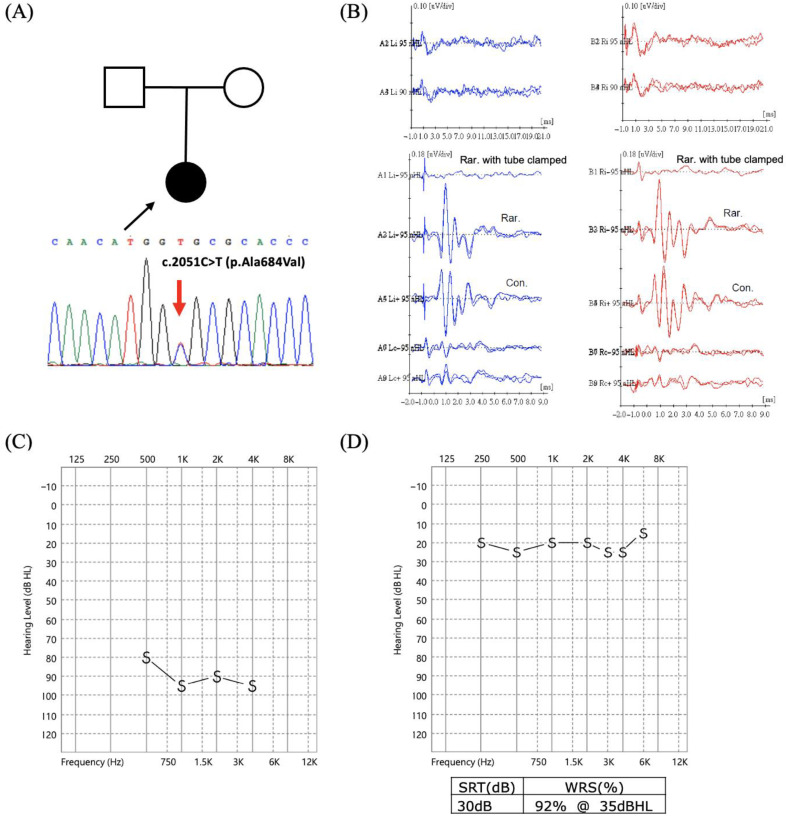
Cochlear implantation outcome in a patient with *WFS1* c.[2051C>T];[2051=]. (**A**) The heterozygous *WFS1* c.2051C>T (p.A684V) variant identified in the proband. (**B**) Cochlear microphonics were recoded despite no wave V identified at 95 dBnHL on auditory brainstem response examinations bilaterally, showing the typical presentation of auditory neuropathy spectrum disorder. (**C**) Preoperative behavioral audiometry reveals profound sensorineural hearing loss with a hearing threshold of approximately 90 dBHL. (**D**) Postoperative behavioral and speech audiometry show ideal cochlear implant-aided hearing threshold and good speech perception. Abbreviations: Con, condensation; Rar, rarefaction; SRT, speech recognition threshold; WRS, word recognition score.

**Figure 2:**
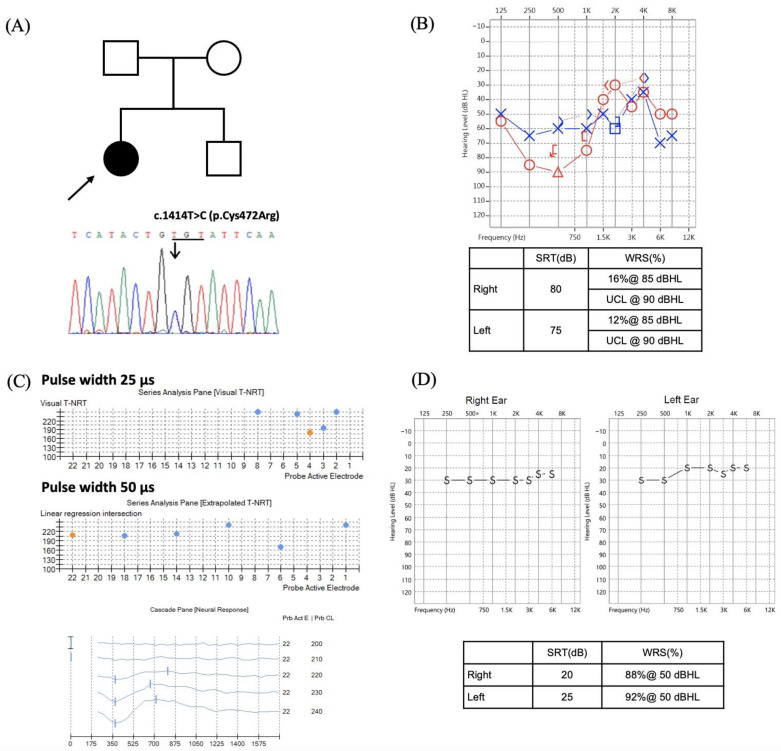
Cochlear implantation outcome in patient with *OPA1* c.[1414T>C];[1414=]. (**A**) The heterozygous *OPA1* c.1414T>C (p.C472R) variant identified in proband. (**B**) Preoperative pure tone audiogram shows bilateral moderate-to-severe hearing loss (~60–80 dBHL) with poor speech discrimination (word recognition scores: 16% on the right and 12% on the left). Red and blue lines represent the hearing thresholds of right and left ear, respectively. (**C**) The electrically evoked compound action potentials were only recordable at high-frequency electrodes (i.e., electrodes #2, 3, 4, 5, and 8) with regular setting but became more robust with an increase in stimulating pulse width from 25 µs to 50 µs. (**D**) Postoperative behavioral and speech audiometry show ideal cochlear implant-aided thresholds bilaterally and good speech perception. Abbreviations: SRT, speech recognition threshold; WRS, word recognition score.

Two patients were identified with rare gene variants, one with *WFS1* c.[2051C>T];[2051=] (Figure 1A) and the other with *OPA1* c.[1414T>C];[1414=] (Figure 2A). The patient with the *WFS1* c.2051C>T (p.A684V) variant was diagnosed with bilateral ANSD at 3 months (Figure 1B), and behavioral audiometry showed profound SNHL (Figure 1C). She underwent cochlear implantation at 2.6 years and exhibited good CI outcomes, with CAP = 6 and SIR = 5 at 3.8 years postoperatively. The CI-aided hearing threshold was 21.3 dBHL with good speech perception (word recognition score (WRS): 92%) (Figure 1D).

A patient with the *OPA1* c.1414T>C (p.C472R) variant presented with slowly progressive hearing loss that deteriorated to moderate-to-severe SNHL in her teens (Figure 2B). However, the speech recognition deteriorated rapidly (WRS = 12–16%). The patient underwent bilateral sequential cochlear implantation. During the surgery, the electrically evoked compound action potentials (ECAPs) were only recorded at high-frequency electrodes with regular settings but became more robust when the stimulating pulse width was increased from 25 µs to 50 µs (Figure 2C). Despite the abnormal ECAPs, the patient showed good auditory and speech performance after cochlear implantation, with a CAP of 7, SIR of 5, and WRS of 88–92% (Figure 2D).

### 3.3. CI Outcomes in ANSD Patients with CND

**Figure 3:**
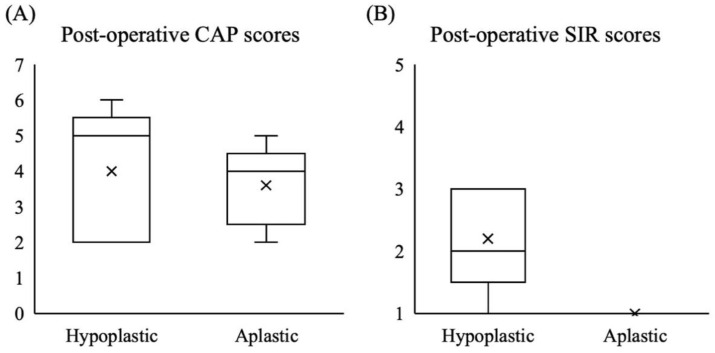
Comparison of the cochlear implantation outcomes in patients with hypoplastic and aplastic cochlear nerves. (**A**) The postoperative Categories of Auditory Performance (CAP) scores were similar between the two groups. (**B**) The postoperative Speech Intelligibility Rating (SIR) scores were significantly better in patients with hypoplastic cochlear nerves than those with aplastic cochlear nerves.

**Figure 4:**
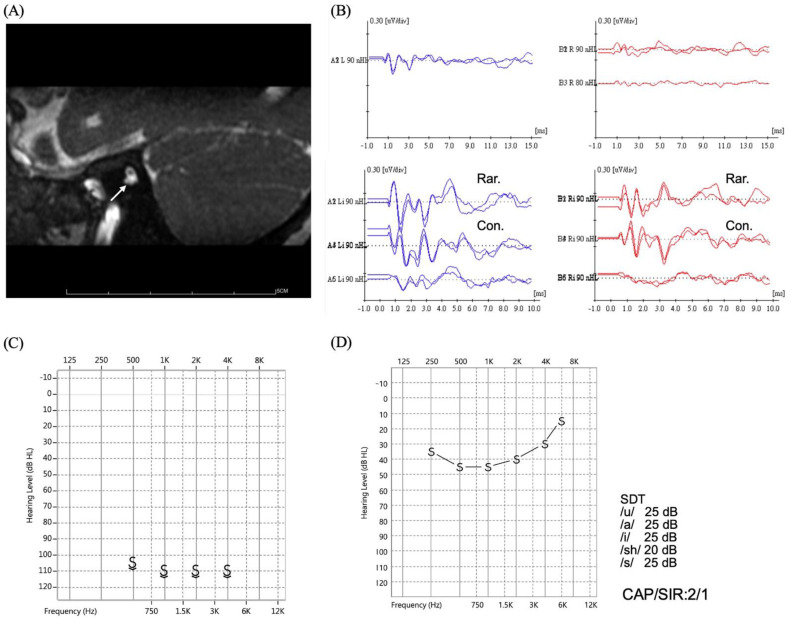
Cochlear implantation outcome in a patient with hypoplastic cochlear nerve. (**A**) Oblique sagittal view of the T2-weighted images on magnetic resonance imaging reveals hypoplastic cochlear nerve (arrow). (**B**) Auditory brainstem responses show no obvious wave V at 90 dBnHL, but cochlear microphonics are recorded. (**C**) The preoperative behavioral thresholds show profound hearing loss (~110 dBHL). (**D**) Poor speech performance after implantation with CAP = 2 and SIR = 1 despite elevated aided hearing threshold (~40 dBHL). Abbreviations: CAP, Categories of Auditory Performance; Con, condensation; Rar, rarefaction; SDT, speech detection threshold; SIR, Speech Intelligibility Rating.

Ten patients were diagnosed with CND based on imaging studies, including five males and five females. Two (20%) patients received bilateral implantations. Of the 12 ears implanted, 5 had aplastic and 7 had hypoplastic cochlear nerves. The hearing thresholds were 100.9 ± 12.1 (80–120) dBHL before cochlear implantation. The average CI-aided hearing threshold in these 10 patients was 38.9 ± 9.3 (25–55) dBHL, which was worse than that in the other groups (Kruskal–Wallis test, *p* < 0.05). In addition, the median CAP and SIR scores at the latest follow-up were 4 (range, 2–6) and 1 (range, 1–3), respectively. Two patients showed relatively favorable CI outcomes with CAP = 5 and SIR = 3 and CAP = 6 and SIR = 2, respectively. Two patients showed improved auditory performance (both CAP = 5) despite poor speech performance (SIR = 2 and 1, respectively) after cochlear implantation. The other six patients with CND showed unfavorable CI outcomes (CAP < 5 and SIR < 3). In general, patients with hypoplastic cochlear nerves showed similar auditory performance with CAP scores (Mann–Whitney U test, *p* = 0.69) but significantly better speech performance with SIR scores (Mann–Whitney U test, *p* = 0.032) than those with aplastic cochlear nerves after receiving CI (Figure 3). Notably, one patient with a hypoplastic cochlear nerve implanted at 1.3 years showed poor sound detection and poor speech development (CAP = 2, SIR = 1) (Figure 4). However, one patient without an identified cochlear nerve on MRI studies presented fair speech recognition despite poor language development (CAP = 5, SIR = 1).

### 3.4. CI Outcomes in ANSD Patients with Indefinite Etiologies

After comprehensive etiological analysis, no specific etiology or risk factors were identified in the six patients. All six patients in this group were male, and two (33.3%) underwent bilateral implantation. The average hearing threshold before cochlear implantation was 97.3 ± 8.7 (90–110) dBHL, and the postoperative CI-aided hearing threshold was 28.3 ± 0.7 (27.5–28.8) dBHL. The median CAP and SIR scores in this group were 5 (range, 2–7) and 4 (range, 1–5) at the latest follow-up, respectively. Three patients had favorable CI outcomes (CAP = 6–7 and SIR = 4–5), while the other three showed unfavorable CI outcomes (CAP = 2–4 and SIR = 1–2).

## 4. Discussion

In this study, we evaluated the CI outcomes of 36 pediatric ANSD patients, including 18 with *OTOF*-related ANSD, 1 with *WFS1*-related ANSD, 1 with *OPA1*-related ANSD, 10 with CND, and 6 without definite etiology. Auditory and speech performance varied according to etiology. Patients with *OTOF*-related, *WFS1*-related, and *OPA1*-related ANSD showed favorable CI outcomes, whereas patients with CND mostly exhibited unfavorable CI outcomes. The CI outcomes in patients with indefinite etiologies were variable, with three patients showing favorable outcomes and the other three unfavorable CI outcomes.

The highly variable outcomes of CI could be a reflection of the diverse pathogenic mechanisms and auditory presentations of ANSD. In a recent review, patients with ANSD demonstrated similar CI outcomes to patients with SNHL, despite the heterogeneity in the etiological background [22]. Still, approximately 25% of pediatric ANSD patients garnered limited benefits from CI [9]. Presynaptic pathologies have been proposed as good prognostic factors for CI, and those with postsynaptic pathologies show variable CI outcomes [7,23]. In this study, we analyzed CI outcomes in patients with ANSD of various etiologies. Our results are generally consistent with those of previous studies; patients with presynaptic pathologies (such as *OTOF*-related ANSD) typically showed good CI outcomes. However, we demonstrated that patients with certain postsynaptic pathologies, such as *WFS1-* and *OPA1-*related ANSD, could also exhibit favorable CI outcomes.

Pathogenic variants in the *OTOF* gene (OMIM:603681), which have been linked to autosomal recessive non-syndromic DFNB9, are the most common genetic cause of pediatric ANSD [24]. Otoferlin, encoded by *OTOF*, is involved in the membrane fusion of synaptic vesicles in the inner hair cells [24,25]. *OTOF*-related ANSD belongs to “presynaptic” ANSD, and the CI outcome is expectedly good in these patients [26,27,28]. In our previous studies, we demonstrated that patients with pathogenic *OTOF* variants always revealed robust ECAPs during cochlear implantation. Postoperatively, these patients usually showed good and rapid improvement in CAP and SIR scores, comparable to those with cochlear SNHL of other genetic causes (such as *GJB2* and *SLC26A4* variants) [29,30]. Before the operation, these patients did not experience spontaneous recovery in hearing thresholds or ABR with age [26]. Given the stable auditory features and favorable CI outcomes, cochlear implantation should be performed in patients with *OTOF* variants whenever indicated without unnecessary delay.

Pathogenic variants of the *WFS1* gene (OMIM:606210) may cause Wolfram syndrome, Wolfram-like syndrome, or autosomal dominant non-syndromic DFNA6/14/38 [31,32,33,34,35]. Wolframin (WFS1), encoded by *WFS1*, is an endoglycosidase H-sensitive membrane glycoprotein localized in the endoplasmic reticulum that is involved in the regulation of intracellular calcium homeostasis [36]. WFS1 is predominantly expressed in the spiral ganglion neuron [37,38], and impaired WFS1 function is related to the “postsynaptic” ANSD. Patients with *WFS1* variants have been reported to have favorable outcomes after cochlear implantation [39,40]. Our patient with the *WFS1* c.2051C>T (p.A684V) variant also exhibited good auditory and speech performances with CI. Notably, Rendtorff et al. reported a family with the c.2051C>T (p.A684V) variant, and CI outcomes were variable [32]. However, the unfavorable CI outcomes observed in this study were ascribed to old implantation age and insufficient postoperative rehabilitation, rather than a genetic factor per se [32].

Pathogenic variants of the *OPA1* gene (OMIM:605290) are the most common cause of autosomal dominant optic atrophy, and patients may present with or without hearing loss [41]. OPA1, encoded by *OPA1*, is a dynamin-related GTPase localized in the mitochondria and is essential for the fusion–fission balance of mitochondria [42]. OPA1 is expressed in multiple sites of cochlea but is predominantly present in the spiral ganglion neurons [43]; thus, ANSD related to defected OPA1 is of “postsynaptic” pathology. Maeda-Katahira et al. reported significant improvements in both auditory and speech performance after cochlear implantation in two patients with *OPA1* variants (c.892A>C and c.1334G>A, respectively) [44]. Santarelli et al. reported CI outcomes in eight patients with different *OPA1* variants (c.344C>T, c.869G>A, c.893G>A, and c.1334G>A), of whom seven showed improved speech perception [45]. Different *OPA1* variants have been associated with varying severities of clinical phenotypes [46]. However, there seems to be no apparent genotype–phenotype correlation in terms of CI outcome, as most patients revealed favorable CI outcomes regardless of the variant.

CND has been regarded as a poor prognostic factor for CI outcomes in both pediatric SNHL and ANSD patients [47,48]. The outcomes vary across different studies; the presence of the cochlear nerve on MRI and the diameter of the cochlear nerve is critical for prognosis [49,50,51]. In our cohort, the CI outcomes were similar to those of a previous study in which those with hypoplastic cochlear nerves showed better outcomes than those with aplastic cochlear nerves. Notably, one patient with an aplastic cochlear nerve presented fair speech recognition despite poor language development. Therefore, the diameter of the cochlear nerve is not the only determinant of CI outcomes in patients with CND. Comprehensive counseling is essential before the operation to establish appropriate parental expectations regarding the prognosis.

The strength of this study lies in the documentation and analyses of CI outcomes in a relatively large cohort of pediatric ANSD patients, for whom a comprehensive etiological work-up was performed and serial behavioral and speech audiometry for at least 1 year were recorded. However, this study has some limitations that deserve further discussion. First, the patients were treated in three CI centers, and there were no unified objective assessment tools for auditory and speech performance among the centers, except for the CAP and SIR scores. Nonetheless, this did not compromise the findings of this study, as both CAP and SIR scores have been confirmed as reliable instruments for measuring CI outcomes [52,53], and we did not observe discrepancies in the CI outcomes in patients with the same etiology between different centers. Second, only three patients with CND and one with indefinite etiology received implantation at less than 2 years of age in this cohort. This may also be a contributing factor to poor outcomes.

Early implantation, especially before 2 years of age, is considered critical for better CI outcomes in pediatric SNHL [54,55]. Based on the findings of this study, we suggest early implantation in pediatric ANSD patients whose etiologies are associated with good CI outcomes whenever the criteria for implantation are fulfilled. Moreover, cochlear implantation is not necessarily contraindicated in patients with postsynaptic pathology, as patients with *WFS1* and *OPA1* variants may also benefit from CI.

## 5. Conclusions

CI-aided hearing thresholds improved in most pediatric ANSD patients, but speech performance varied. Favorable CI outcomes can be anticipated in patients with certain etiologies of ANSD, particularly in those with *OTOF*, *WFS1*, and *OPA1* variants. By contrast, patients with CND have suboptimal outcomes. Identifying the etiologies in ANSD patients is crucial before surgery and can aid in prognosis prediction and counseling for families.

## Figures and Tables

**Table 1:** Subjects with confirmed genotype in this study.

Genotype	Cases (N)
*OTOF*	
*OTOF* c.[1498C>T];[5098G>C]	1
*OTOF* c.[2521G>A];[5098G>C]	2
*OTOF* c.[3704_3719del];[5098G>C]	2
*OTOF* c.[3864G>A];[5098G>C]	1
*OTOF* c.[4030C>T];[5098G>C]	1
*OTOF* c.[4961-1G>A];[5098G>C]	1
*OTOF* c.[5000C>A];[5098G>C]	1
*OTOF* c.[5098G>C];[5098G>C]	7
*OTOF* c.[5098G>C];[5203C>T]	1
*OTOF* c.[5098G>C];[5566C>T]	1
Other rare genes	
*WFS1* c.[2051C>T];[2051=]	1
*OPA1* c.[1414T>C];[1414=]	1

**Table 2:** Basic characteristics of patients with auditory neuropathy spectrum disorder in different groups.

	*OTOF* Variants N = 18	Rare Gene Variants ^a^ N = 2	CND N = 10	Indefinite N = 6	Total N = 36	*p* Value
Sex (M:F)	13:5	0:2	5:5	6:0	24:12	0.034 ^b^
Age at implantation of the first ear (y), mean ± SD [range]	2.8 ± 1.3 [1–5.5]	9.0 ± 9.1 [2.6–15.4]	3.1 ± 1.7 [1.3–6.2]	6.6 ± 10.4 [1.7–27.7]	3.9 ± 4.9 [1–27.7]	0.734 ^c^
Preoperative hearing thresholds (dBHL), mean ± SD [range]	88.2 ± 14.5 [57.5–106.3]	66.3 ± 20.6 [53.8–90]	100.9 ± 12.1 [80–120]	97.3 ± 8.7 [90–110]	91.2 ± 15.9 [53.8–120]	0.022 ^c^
Bilateral implantation (N)	9	1	2	2	14	0.457 ^b^
Follow up (y), mean ± SD [range]	4.3 ± 2.4 [0.8–7.6]	3.2 ± 0.9 [2.6–3.8]	3.5 ± 4 [1–13.8]	4.3 ± 4.3 [0.6–10.1]	4.0 ± 3.1 [0.6–13.8]	0.707 ^c^

^a^ Rare gene variants including variants in *WFS1* and *OPA1,* ^b^ Fisher’s exact test, ^c^ Kruskal–Wallis test. Abbreviations: CND, cochlear nerve deficiency; SD, standard deviation.

**Table 3:** Cochlear implantation outcomes in patients with auditory neuropathy spectrum disorder of different etiologies.

	*OTOF* Variants N = 18	Rare Gene Variants ^a^ N = 2	CND N = 10	Indefinite N = 6	Total N = 36	*p* Value
CI-aided hearing thresholds (dBHL), mean ± SD [range]	25.6 ± 4.1 [20–35]	20.8 ± 1.9 [18.8–22.5]	38.9 ± 9.3 [25–55]	28.3 ± 0.7 [27.5–28.8]	28.3 ± 7.8 [18.8–55]	<0.001 b
CAP score, median [range]	7 [5–7]	7 [6,7]	4 [2–6]	5 [2–7]	6 [2–7]	0.021 b
SIR score, median [range]	5 [2–5]	5 [5]	1 [1–3]	4 [1–5]	4 [1–5]	0.006 b

^a^ Rare gene variants including variants in *WFS1* and *OPA1*, ^b^ Kruskal–Wallis test, Abbreviations: CAP, Categories of Auditory Performance; CI, cochlear implant; CND, cochlear nerve deficiency; SIR, Speech Intelligibility Rating.

## Data Availability

The data presented in this study are available on request from the corresponding author. The data are not publicly available due to their containing information that could compromise the privacy of research participants.
